# Blood bronchial mucus with Dirofilaria immitis adult worms after the treatment with doxycycline and moxidectin: a rare case presentation

**DOI:** 10.1017/S0031182026101577

**Published:** 2026-03

**Authors:** Larissa Leykman da Costa Nogueira, Bruno Vinicios Silva de Araújo, João Marcelo Azevedo de Paula Antunes, Moacir Bezerra de Andrade, Rafael Antonio Nascimento Ramos, Norma Labarthe, Renata Pimentel Bandeira de Melo, Leucio Câmara Alves

**Affiliations:** 1 Department of Animal Science, Federal Rural University of the Semi-Arid Region (UFERSA), Rio Grande do Norte, Brazil; 2 Department of Veterinary Medicine, Federal Rural University of Pernambuco (UFRPE), Recife, PE, Brazil; 3 Department of Veterinary Pathobiology, College of Veterinary Medicine and Biomedical Sciences, Texas A&M University, College Station TX 77843, USA; 4 National School of Public Health, Oswaldo Cruz Foundation (ENSP/FIOCRUZ), Rio de Janeiro, Brazil; 5 Oswaldo Cruz Foundation (FIOCRUZ), Rio de Janeiro, Brazil

**Keywords:** bronchial expectoration, canine cardiopulmonary dirofilariosis, hemoptysis, molecular diagnosis

## Abstract

Heartworm disease is a serious acquired parasitic infection caused by *Dirofilaria immitis* that can cause severe lung manifestation, heart failure, and other organ damage in dogs. We hereby present a case of a seven-year-old, intact male which was submitted to treatment of heartworm disease with doxycycline and a single dose of injectable moxidectin. Three weeks later the dog presented an acute coughing, hemoptysis and marked dyspnea and was admitted to the Hospital Veterinário Popular de Mossoró, a private veterinary hospital located in Mossoró, Rio Grande do Norte, Brazil. Some hours after the admission, the dog showed a severe coughing episodes with progressive expectoration and subsequently profuse bloody discharge. At this time, two live worms were coughed out. The nematodes specimens (1 male and 1 female) were collected from bronchial mucus, and morphologically and molecularly identified as *D. immitis*. A fragment of 635 bp of the cytochrome oxidase C subunit 1 gene was sequenced and deposited at the GenBank database (Accession number: PV729978). The treatment of heartworm disease can be complex, and the veterinarian must evaluate each case individually to determine the best treatment protocol. Veterinarian practitioners must be aware about the risk of this presentation and intervene properly to keep dogs’ welfare.

## Graphical abstract

**Figure:**
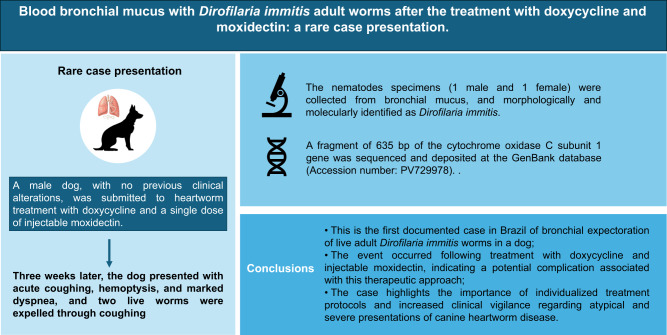

## Introduction

*Dirofilaria immitis* is a filarial nematode commonly referred to as heartworm. It is transmitted to a variety of vertebrate hosts, primarily dogs, but also cats, wild animals, and occasionally humans through the bite of infected mosquitoes (Simón *et al.,* 2012; Dantas-Torres and Otranto, 2020; Alsarraf *et al.,* 2023).

In most cases, heartworm disease in dogs presents as a subclinical infection, with no apparent clinical signs (American Heartworm Society, 2024). However, in advanced stages, affected dogs may exhibit coughing, dyspnea, episodic collapse, anorexia, weight loss, ascites, anemia and thrombocytopenia, which may evolve to a potentially fatal condition (McCall *et al.,* 2008; Simón *et al.,* 2012; Bendas *et al.,* 2022; Lemos *et al.,* 2022).

Adult worms reside primarily in the pulmonary arteries, where they contribute to the development of pulmonary hypertension followed in the most severe cases by right-sided congestive heart failure (Serrano-Parreño *et al.,* 2017). This condition leads to reduced perfusion of peripheral organs due to cardiopulmonary impairment, resulting in secondary damage to the lungs, liver, kidneys, and overall deterioration of the animal’s health (Pasca *et al.,* 2012).

The diagnosis of heartworm disease in dogs is primarily based on the detection of circulating antigens and microfilariae, along with clinical evaluation and diagnostic imaging findings (Nelson *et al.,* 2018; Smith *et al.,* 2024).

As melarsomine dihydrochloride is not available in Brazil, the treatment of canine heartworm disease typically involves a combination of macrocyclic lactones and antibiotics, particularly doxycycline, as part of an adulticidal protocol (Moraes-da-Silva *et al.,* 2016; Nelson *et al.,* 2020; Jacobson and DiGangi, 2021).

Although there are no published studies determining the prevalence of *D. immitis* in dogs in the state of Rio Grande do Norte, documented occurrences of the disease have been reported in the region (Moraes *et al.,* 2021; Silva *et al.,* 2023). These findings indicate that the parasite is indeed circulating in the state, highlighting the potential risk of transmission to the local human population.

Canine heartworm disease is a parasitic condition of significant clinical relevance in veterinary medicine, particularly in endemic regions such as Brazil. While many infections remain asymptomatic or present with mild clinical signs, severe and atypical manifestations can occur and require careful clinical attention. This report aims to describe a rare case of expectoration of blood-tinged bronchial mucus containing adult *D. immitis* worms following treatment with doxycycline and moxidectin.

## Case report

A seven-year-old intact male Yorkshire terrier from Mossoró, Rio Grande do Norte, Brazil (05°11’16.8’’ S, 37°20’38.4’’ W), was referred to the veterinary cardiology service in July 2024 following the echocardiographic identification of structures consistent with *D. immitis*. The dog was asymptomatic, with a history of intermittent coughing.

On clinical examination, the patient presented with a systolic blood pressure of 140 mmHg, a heart rate of 120 bpm, and a grade III/VI tricuspid murmur. Electrocardiographic evaluation revealed sinus arrhythmia with a single ventricular premature complex (VPC). A rapid antigen test for *D. immitis* (Alere® Ag, Alere Inc., Waltham, MA, USA) scored positive. Treatment was initiated with oral doxycycline (10 mg/kg, BID for 30 days) and a subcutaneous injection of sustained-release moxidectin (ProHeart® SR-12; 0.05 mL/kg). The owner was advised to implement strict exercise restriction for the duration of the treatment protocol.

Nine days later, the patient returned with signs of apathy and mild dyspnea. Thoracic radiographs revealed diffuse inflammatory bronchopneumopathy and pulmonary hypervascularization. Prednisolone therapy was initiated, along with nebulization using isotonic saline.

Three weeks later, the dog presented with acute onset of coughing, hemoptysis, and marked dyspnea, accompanied by muffled pulmonary sounds on auscultation. The patient was hospitalized and treated with oxygen supplementation, butorphanol (0.02 mg/kg, QID), dexamethasone (0.5 mg/kg, SID), and dipyrone (25 mg/kg, BID).

**Figure 1.**
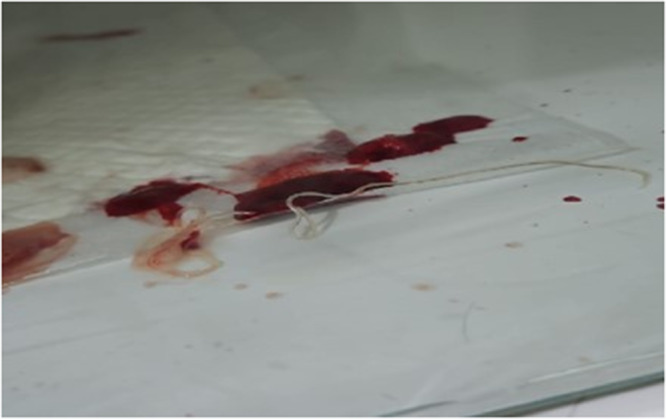
Filarial worm expelled by the dog following an episode of hemoptysis. Figure 1 long description.

Within hours, the dog experienced repeated episodes of intense coughing with progressive expectoration, beginning with foamy, whitish mucus and evolving into copious bloody discharge. During one of these episodes, two live nematodes were expelled orally. The specimens consisted of one male and one female, measuring 14 cm and 30 cm in length, respectively, with an approximate diameter of 3 mm (Figure 1).

Due to the severity of hemorrhagic complications, tranexamic acid (25 mg/kg, TID) was administered. The patient’s respiratory status improved gradually. Follow-up thoracic radiographs revealed persistent diffuse bronchopneumopathy, enlargement of the pulmonary arteries, and prominence of the pulmonary trunk. Doppler echocardiography confirmed pulmonary valve insufficiency.

Additionally, mild pulmonary hypertension (estimated systolic pulmonary arterial pressure of 38 mmHg), thickening of the tricuspid valve, moderate right-sided cardiac chamber remodeling, and the presence of filarial worms within a branch of the right pulmonary artery were identified. Three days after hospitalization, the patient exhibited stable clinical parameters with no recurrence of clinical signs.

Both of worms was observed morphologically and identified as *D. immitis* according Khanmohammadi et al. (2020). Fragments of adult worms were subjected to DNA extraction using the commercial NucleoSpin Tissue kit (Macherey-Nagel GmbH & Co. KG, Düren, Germany), following the manufacturer’s protocol. The extracted genomic DNA was quantified by spectrophotometry using a NanoDrop™ Lite instrument (Thermo Fisher Scientific, Waltham, MA, USA), and samples were stored at – 20 °C until polymerase chain reaction (PCR) analysis.

A 635 bp fragment of the mitochondrial cytochrome C oxidase subunit I (COI) gene, specific to filarial nematodes, was amplified by conventional PCR using the primers COIintF and COIintR as described by Casiraghi et al. (2001). PCR amplification was performed under the following cycling conditions: an initial denaturation at 95 °C for 2 minutes; 40 cycles of denaturation at 95 °C for 45 seconds, annealing at 52 °C for 45 seconds, and extension at 72 °C for 90 seconds; followed by a final extension at 72 °C for 5 minutes.

Each PCR reaction was carried out in a final volume of 15 μL, containing 6.75 μL of GoTaq® G2 Green Master Mix (Promega Corporation, Madison, WI, USA), 1 μL of each primer (10 μM), 3.25 μL of DEPC-treated water, and 3 μL of template DNA. A positive control consisting of DNA from a previously confirmed positive sample was included, along with DEPC-treated water as a negative control.

The PCR amplicons were subjected to electrophoresis on a 1.5% agarose gel prepared in 1 × TAE buffer (Tris-acetate-EDTA), with Blue Green (LCG Biotecnologia Ltda., Cotia, SP, Brazil) used as the nucleic acid stain. A 100 bp molecular weight marker (GeneDirex®, Taoyuan, Taiwan) was employed to estimate the size of the amplified fragments, following the manufacturer’s guidelines. After electrophoresis, the gel was visualized under ultraviolet (UV) illumination using a transilluminator equipped with a digital imaging system.

PCR products were purified using a silica membrane-based protocol provided by the commercial PureLink™ Quick Gel Extraction and PCR Purification Combo Kit (Thermo Fisher Scientific, Waltham, MA, USA), according to the manufacturer’s instructions. DNA concentration and purity were assessed via spectrophotometry (NanoDrop™ Spectrophotometer, Thermo Fisher Scientific, Waltham, MA, USA).

Sequencing was conducted at the Human Molecular Genetics Laboratory of the Federal University of Pernambuco using the Sanger method with forward and reverse primers. Resulting sense and antisense chromatograms were quality-trimmed and assembled using BioEdit software (version 7.2.5). The consensus sequences were aligned and compared to entries in the GenBank® nucleotide database using the BLAST® algorithm to confirm sequence identity. The partial sequence of the cytochrome c oxidase subunit I (COI) gene obtained in this study was deposited in the GenBank database under the accession number PV729978.

## Discussion

Most *D. immitis* infections are either asymptomatic or present with mild clinical signs, such as intermittent coughing, as observed in the present case (Vieira *et al.,* 2014; Pietrzak *et al.,* 2024).

Thoracic radiography proved to be a valuable diagnostic tool for detecting pulmonary alterations consistent with heartworm infection and for guiding supportive therapeutic interventions. However, thoracic radiographs may have limited sensitivity for identifying early or subtle vascular changes and may underestimate thromboembolic involvement, particularly when concurrent pulmonary disease is present (Falcón-Cordón *et al.,* 2024).

In such cases, computed tomography (CT), especially CT angiography, can provide higher-resolution visualization of pulmonary arterial branches and parenchymal lesions, serving as a useful complementary imaging modality (Jung *et al.,* 2010). Nevertheless, in the present case, radiographic findings were informative and underscored that even clinically asymptomatic animals may exhibit significant pulmonary pathology during the subclinical stages of the disease (Lima *et al.,* 2025). Clinical findings such as tricuspid murmur and pulmonary valve regurgitation were considered common alterations in dogs infected with *D. immitis* (Lemos *et al.,* 2022).

Ectopic migration of *D. immitis* to anatomical locations beyond the heart and pulmonary arteries has been documented, with adult worms identified in the abdominal cavity, central nervous system, subcutaneous tissue, and ocular conjunctiva. However, the underlying pathophysiological mechanisms facilitating such aberrant migration remain poorly elucidated (Kotani *et al.,* 1975; Goh *et al.,* 2023; Barnett *et al.,* 2025).

Although the expectoration of adult *D. immitis* worms has not been previously reported in the literature, the clinical signs observed in this patient namely coughing, hemoptysis, tachypnea and dyspnea are consistent with pulmonary thromboembolism secondary to the presence of intravascular adult parasites (Ames and Atkins, 2020; Bendas *et al.,* 2022; American Heartworm Society, 2024).

Rishniw et al. (2012) reported hemoptysis associated with *D. immitis* infection in five dogs treated with different therapeutic agents for heartworm disease. Hemoptysis, or the expectoration of blood, has been described in cases of heavy heartworm burden in dogs (Greenway *et al.,* 2019; Alberigi *et al.,* 2021). In the present case, we report the first documented occurrence in Brazil of blood-tinged bronchial mucus containing an adult *D. immitis* worm following treatment with doxycycline and moxidectin.

The molecule choice to treat heartworm infections in dogs is unavailable in many countries where heartworm is endemic (Dantas-Torres *et al.,* 2023) and the combination of doxycycline and macrocyclic lactone as ivermectin or moxidectin are used (Moraes-da-Silva *et al.,* 2016; Nelson *et al.,* 2020; Jacobson and DiGangi, 2021).

Although coughing and vomiting in dogs can result from a variety of underlying conditions, vomiting has specifically been reported during doxycycline therapy (Schulz *et al.,* 2011).

The exact mechanism underlying filarial expectoration remains unclear. However, it is hypothesized that rupture of the capillaries within the alveolar-capillary barrier may allow the translocation of adult worms typically located in the pulmonary arteries, into the pulmonary alveoli, bronchioles, and the secondary, tertiary, and primary bronchi. This displacement may be facilitated or exacerbated by the cough reflex, which is frequently observed in affected individuals. While the rupture of a single alveolar capillary is unlikely to be fatal, it may initiate a cascade of inflammatory responses, including pneumonia, bronchiolitis, bronchitis, and pleuritis, which can develop in the days following the initial vascular injury.

Rishniw et al. (2012) described hemoptysis associated with *D. immitis* infection, demonstrating that substantial pulmonary endothelial injury can result in bloody expectoration even in the absence of a high parasitic burden. These observations support the hypothesis advanced in the present report that expulsion of an adult worm via the airways is more likely attributable to disruption of the capillary–alveolar barrier and subsequent passive translocation of intravascular parasites into the respiratory tract, potentially facilitated by coughing, rather than to erratic migratory behavior. Although erratic migration cannot be entirely excluded, the available evidence favors secondary translocation following vascular rupture as the primary mechanism of airway elimination.

Despite the occurrence of marked hemorrhage associated with worm expectoration, the condition was promptly stabilized, and the patient was discharged following three days of hospitalization. The caregiver was advised regarding the potential for long-term pulmonary sequelae secondary to parasitic infection. The authors emphasize that both hemoptysis and hematemesis, even when accompanied by the presence of adult parasites, may occur in cases of canine dirofilariosis and do not necessarily signify a severe or life-threatening stage of the disease.

## Figure 1 Long description

Title text: Blood bronchial mucus with Dirofilaria immitis adult worms after the treatment with doxycycline and moxidectin: a rare case presentation. Section heading: Rare case presentation. An illustration shows lungs and a dog silhouette. Text: A male dog, with no previous clinical alterations, was submitted to heartworm treatment with doxycycline and a single dose of injectable moxidectin. A downward arrow connects to the next text block: Three weeks later, the dog presented with acute coughing, hemoptysis and marked dyspnea and two live worms were expelled through coughing. A separate text block includes a microscope icon and a DNA icon. Text: The nematodes specimens (1 male and 1 female) were collected from bronchial mucus and morphologically and molecularly identified as Dirofilaria immitis. Text: A fragment of 635 bp of the cytochrome oxidase C subunit 1 gene was sequenced and deposited at the GenBank database (Accession number: PV729978). Section heading: Conclusions. Bullet points: This is the first documented case in Brazil of bronchial expectoration of live adult Dirofilaria immitis worms in a dog; The event occurred following treatment with doxycycline and injectable moxidectin, indicating a potential complication associated with this therapeutic approach; The case highlights the importance of individualized treatment protocols and increased clinical vigilance regarding atypical and severe presentations of canine heartworm disease.

Navigate back to Figure 1.
